# Bortezomib alleviates antibody‐mediated rejection in kidney transplantation by facilitating Atg5 expression

**DOI:** 10.1111/jcmm.16998

**Published:** 2021-11-03

**Authors:** Hong Cheng, Bin Xu, Lijie Zhang, Yi Wang, Ming Chen, Shuqiu Chen

**Affiliations:** ^1^ Department of Urology Zhongda Hospital Affiliated to Southeastern China University Nanjing China; ^2^ Department of Urology Affiliated Hospital of Nantong University Nantong China

**Keywords:** antibody‐mediated rejection, Atg5, bortezomib, kidney transplantation, rat model

## Abstract

Antibody‐mediated rejection (AMR) is one of the most dominant mechanisms responsible for the loss of kidney grafts. Previous researches have shown that donor‐specific antibodies (DSAs) are the major mediators of AMR. In order to prolong the survival time of grafts, it is vital to reduce the incidence of AMR and inhibit the generation of DSAs. We established an animal model of AMR by performing kidney transplantation in pre‐sensitized rats. Then, we investigated the effect of bortezomib (BTZ) on AMR. We found that BTZ could reduce the serum level of DSAs and alleviate post‐transplantation inflammation in peritubular capillaries (PTCs) and glomeruli, which was demonstrated by the reduction of C4d and IgG deposition in PTCs, and the reduced number of B cell and plasma cell in peripheral blood and the transplanted kidney (*p* < 0.05). Our results also suggested that BTZ increased the number of regulatory T cell (Treg) and significantly reduced the proportion of T helper (Th17) cell (*p* < 0.05). Besides, BTZ induced the significant upregulation of anti‐inflammatory cytokines but downregulated pro‐inflammatory cytokines (*p* < 0.05). After dealing with Atg5 siRNA‐lentivirus, the effect of BTZ alleviating AMR was reversed and Th17/Treg proportions were also significantly modulated. Collectively, these findings show that BTZ slows down the process of AMR and Atg5 may be the key mechanism. Furthermore, Atg5 silencing results may be demonstrated that Atg5 alleviated AMR by modulating the ratio of Th17/Treg.

## 1 INTRODUCTION

Kidney transplantation is well established as the optimal therapy for patients with end‐stage renal diseases and can improve life quality for such patients.^1^ Allograft rejection following renal transplantation, including acute and chronic rejection, is the most dominant threat for the long‐term survival of a transplanted kidney.^2^ Immunity can be classified into cellular immunity and humoral immunity, according to the specific mechanisms involved.^3^ Increasing studies have found that antibody‐mediated humoral rejection plays a significant role in the tissue grafts loss.^4^ Despite the fact that numerous treatments are available for T cell–mediated rejection, the options for treating AMR are very limited.^5^ Current strategies to reduce the incidence of AMR predominantly involve removing antibodies and blocking the activation of complement, such as plasmapheresis, intravenous immunoglobulin and rituximab.^6^ However, these strategies are limited due to severe complications and high cost. Therefore, there is an urgent need to develop new methods to control AMR.

Regulatory T cells (Treg) is specific cell that has the ability to regulate immune function. It is well established that Treg is one of the key mechanisms used to regulate inappropriate or excessively strong immune response.^7^ Treg can actively inhibit T‐cell function and maintain immune homeostasis in the body. Studies have shown that Foxp3^+^ Treg can inhibit T cell by producing immunosuppressive factors such as IL‐10, IL‐35 and TGF‐β.^8^ Treg also plays an important role in the pathogenesis of different diseases, including autoimmune disease, chronic inflammation and the development of tumours.^9^, ^10^, ^11^ Clinical studies have reported that the increased expression of Treg was associated with a reduced incidence of AMR.^12^, ^13^ In recent years, researches have shown that Th17/Treg imbalance plays a important role in the pathogenesis of diseases, such as autoimmune diseases and asthma.^14^, ^15^, ^16^, ^17^ In mice heart transplantation, it was reported that the effect mediated by Th17/Treg imbalance was related to the occurrence of graft rejection.^18^

Bortezomib (BTZ) was the first clinical proteasome inhibitor and has been approved for the treatment of multiple myeloma, a malignant plasma cell (PC) disorder.^19^ BTZ was reported to inhibit the growth of malignant tumour cell by blocking the nuclear factor NF‐κB signalling pathway and alleviate fibrosis of the skin, lung and kidney.^20^, ^21^, ^22^, ^23^ In addition, BTZ has been proposed as a candidate for ABMR treatment.^24^, ^25^ The mechanism of AMR is known to be mediated by alloantibodies, and BTZ can inhibit the production of such antibodies. However, whether BTZ can exert effect on AMR by Treg remains untested. Therefore, in the current study, we explored the effect of BTZ on AMR and investigated the potential mechanisms underlying these effects.

## 2 MATERIALS AND METHODS

### 2.1 Ethics statement

All animal experiments were conducted in the Animal Core Facility of Nanjing Medical University and were performed in accordance with National Health Organization and Nanjing Medical University Institutional Ethical Guidelines for animal experiments (Experimental ethics number: IACUC1601140‐1).

### 2.2 Reagents

BTZ (0.2 mg/kg; Selleck Chemicals) was injected intravenously into recipient rats every day following kidney transplantation. Before the experiment, we divided BTZ into three concentration gradients for pre‐experiment, and finally confirmed the best concentration. Rat Atg5 siRNA‐lentivirus (Atg5 siRNA) (sc‐41445) were acquired from Santa Cruz Biotechnology. The antibodies used for Western blot (WB) (Atg5, Atg7 and Atg12) were purchased from Cell Signaling Technology. The antibodies used for immunohistochemistry (anti‐CD19 and anti‐CD138) were purchased from Abcam. The antibodies used for immunofluorescent staining were anti‐rat C4d antibody (American Research Products, Belmont), FITC‐conjugated goat anti‐rat IgG antibody (Jackson ImmunoResearch). Several antibodies were used for flow cytometric analysis: FITC‐CD4, APC‐CD25, PE‐FOXP3 and PE‐IL‐17A (Thermo Fisher Scientific); APC‐CD19 (Beijing Biosynthesis Biotechnology Co. Ltd.,); and APC‐CD138 (Abcam).

### 2.3 Animals

Lewis (LEW) (RT1^u^) rats were used as donors, and Brown Norway (BN; RT1^l^) rats were used as recipients. These animals were purchased from Beijing Vital River Laboratory Animal Technology Co. Ltd. All rats were male, 6 weeks of age and weighed between 180 and 220g.

### 2.4 Skin transplantation and renal transplantation

Recipients and donors were fasted for 12 h before kidney transplantation or skin transplantation; they were also prevented from drinking water for 8 h prior to surgery. Pieces of skin were removed from LEW rats and transplanted on to BN rats. Full‐thickness skin grafts (approximately 1.5 cm × 1.5cm) were taken from the hackles of LEW rats and then transplanted onto the hackles of BN rats. Once the skin was removed from the donor LEW rats, it was wrapped with gauze and placed in ice water containing penicillin (8 × 104 u/ml). Blood samples were then obtained from the BN rats, and serum samples were prepared by centrifugation. We then tested the serum concentrations of DSAs after skin transplantation at 1, 3, 7, 10, 14, 21, 28 and 35 days, respectively. Kidney transplantation was performed when the level of DSAs reached peak values. After anaesthesia, the LEW rats were fixed, disinfected and incisions were made along the middle abdominal line. Next, we fully exposed the abdominal aorta, inferior vena cava, renal artery and renal vein; these vessels were carefully isolated by light microscopy. The kidney of donor animals was perfused, placed in perfusion fluid, and then stored in a refrigerator at 4°C. Similarly, we also visualized and separated the renal artery, renal vein and ureter of BN. The renal artery and renal vein were then anastomosed end‐to‐end, and the ureter was embedded into the bladder. After the removal of the contralateral kidney, the abdomen was closed and disinfected. We obtained further serum and transplant kidney on day 4 after kidney transplantation. Serum samples were frozen at −80°C to await analysis. Every transplanted kidney was divided into two parts. One was partially filled with RNAkeeper (Vazyme Biotech Co. Ltd) solution and then stored at −80°C while another part was kept in 4% paraformaldehyde.

### 2.5 Experimental groupings

Our experiments featured four groups of rats: a normal group (non‐treated BN rats); a skin group (STx) featuring BN rats on day 14 after skin transplantation; a kidney transplantation group (KTx) featuring rats that underwent renal transplantation; and an AMR group featuring BN rats which underwent renal transplantation after skin grafting. Every group have at least 5 rats samples. Kidneys were harvested on day 4 after kidney transplantation. Our aim was to use AMR model to investigate the effect of BTZ. Similarly, in this research, every group have at least 5 rats samples. BN rats were injected with 0.2 mg/kg of normal saline (once daily by tail vein injection) after receiving an open/closed operation; these formed a Con‐Veh group. Another group of BN rats were given 0.2 mg/kg of BTZ (once daily) after receiving an open/closed operation; these formed a Con‐BTZ group. The AMR‐Veh group featured pre‐sensitized BN rats that were given 0.2 mg/kg of normal saline (once daily) following kidney transplantation. While the final group, the AMR‐BTZ group, featured pre‐sensitized BN rats that were given 0.2 mg/kg of BTZ (once daily) following kidney transplantation.

### 2.6 The detection of circulating DSAs and converting data to molecules of equivalent soluble fluorochrome (MESF) units

Spleen lymphocytes were extracted from normal LEW and counted under aseptic conditions. Serum samples of BN rats were incubated with spleen lymphocytes at room temperature for 30 min. Cells were then washed and incubated with fluorescein isothiocyanate (FITC)‐labelled anti‐rat IgG antibody (BD Biosciences) at room temperature for 30 min. Cells were then analysed by flow cytometry (FCM) (Beckman DxFLEX, Beckman), and mean fluorescence intensity (MFI) was measured to determine the serum level of DSA. The Quantum^TM^ MESF Kit (Bangs Laboratories) is supplied with one blank microsphere population and four microsphere populations that are surface‐labelled with increasing concentrations of a specified fluorochrome. These populations are calibrated in MESF units. The five microsphere populations were tested by the Beckman DxFLEX FCM; data were analysed by downloading specific software from www.bangslabs.com. MFI was then converted to MESF units; this practice added internal parameters to the MFI data and increased reliability.

### 2.7 Histopathology

Kidney grafts were taken from the recipients at a fixed time point after transplantation. Allografts were harvested, formalin‐fixed, embedded in paraffin and stained in haematoxylin and eosin (H&E) and periodic acid‐Schiff (PAS). Pathological sections were then observed using a Nikon E100 microscope (Nikon) and investigated for the characteristic features of AMR, including PTC inflammation, glomerulitis and intimal arteritis, as described by the Banff 2013 guidelines.

### 2.8 Immunohistochemistry and immunofluorescence

For immunohistochemistry, renal tissue was fixed with 4% paraformaldehyde, embedded in paraffin and then sectioned. Tissue sections were placed into a box and then heated in a microwave oven for antigen retrieval. Then, we blocked endogenous peroxidase by treating the sections with 3% hydrogen peroxide. Sections were then incubated with a primary antibody followed by an appropriate secondary antibody. Antibody binding was then visualized by DAB treatment. The nuclei were then stained, and the sections were dehydrated. Finally, the sections were mounted on glass slides for analysis. For immunofluorescent analysis, renal tissue was frozen and then embedded in paraffin. In order to observe the deposition of C4d, frozen sections were incubated with rabbit an anti‐C4d antibody (diluted 1:50 in PBS) for 30 min at room temperature. The sections were then washed four times in PBS and then incubated with FITC goat anti‐rabbit IgG. To investigate the deposition of IgG in tissue grafts, we stained 4 mm cryosections with FITC‐conjugated goat anti‐rat IgG. Image‐pro Plus software (version 6.0) was then used to evaluate the expression CD19 CD138 C4d deposition and IgG deposition.

### 2.9 The detection of cytokines in serum and supernatants by enzyme‐linked immunosorbent assays (ELISAs)

Samples of serum and supernatant were acquired, processed and immediately frozen at −80°C to await analysis. The level of cytokines in these samples was then determined using a range of commercial ELISA kits: TNF‐α, IL‐17A, TGF‐β and IL‐10 (Multi Sciences, Hangzhou, China); and IL‐35 (Bioswamp). All assays were carried out in accordance with the manufacturer's guidelines. ELISA data were detected using a Tecan Sunrise infinite M200 PRO platereader (Tecan, Switzerland).

### 2.10 Flow cytometry

First, Red Blood Cell (RBC) Lysis Buffer (Xiansheng Biotechnology Co. Ltd,) was added to whole blood samples to lyse the RBCs. For CD19 and CD138 flow analyses, CD45R also needed to be labelled; appropriate antibodies were incubated with the lyzed blood samples and then detected by a Beckman DxFLEX Flow cytometry (FCM). A CD19 antibody was added and incubated on ice for 45 mins prior to fixation and permeabilization (Thermo Fisher Scientific). Cells were then washed and detected by a Beckman DxFLEX FCM. For the analysis of CD4 and CD25, antibodies were added and incubated on ice for 45 mins prior to fixation and permeabilization (Thermo Fisher Scientific). In addition, 2% normal Sprague‐Dawley (SD) rat serum was used to block cells for 15 mins before incubation for 30 min with an antibody raised against FOXP3. Cells were then washed and analysed by a Beckman DxFLEX FCM. For the analysis of Th17, a CD4 antibody was added and incubated on ice for 45 mins. A Cell Treatment Reagents (Thermo Fisher Scientific, Shanghai, China) Kit was used to deal with for 5–18 h and a Cell Fixation & Permeabilization Kit (Cat. No. FP0050, Formex Biotechnology Co. Ltd) was used to fix and permeabilize cells. An IL‐17A antibody was added and incubated for 15 mins at room temperature. Cells were then washed and detected by a Beckman DxFLEX FCM.

### 2.11 Western blotting

Kidney tissue was used to extract proteins from transplanted kidney tissue. Protein concentration was then determined using a BCA Protein Assay Kit (Pierce). All samples were then separated by 10% sodium dodecyl sulphate polyacrylamide gel electrophoresis (SDS‐PAGE) and then transferred to polyvinylidene fluoride (PVDF, Millipore) membranes. Membranes were then blocked for 60 min with 5% skimmed milk at room temperature and were then incubated overnight at 4°C with antibodies against Atg5 and β‐actin. Membranes were then washed three times in TBST buffer (20 mmol/L Tris‐buffered saline and 0.1% Tween 20), and then incubated for 1h at 37°C with a peroxidase (HRP)‐conjugated secondary antibody. Immunoblotted proteins were then carried out by ImageJ (National Institutes of Health).

### 2.12 Transfer of CD4+ T cells into rat

5 × 10^6 CD4+ T cells were isolated from the spleens of rats and treated with Atg5 siRNA or BTZ for 48 h. Then, the cells injected from caudal veins into Rag1−/− rats 30 min before being subjected to kidney transplantation.

### 2.13 Statistical analysis

Each experiment was repeated at least three times. GraphPad Prism 8.0 software was used for all data analysis. Data are presented as means ± standard deviation. The Student's t test was used to compare differences between groups. The log‐rank test was used to compare graft survival. Differences in which *p* < 0.05 were considered to be statistically significant.

## 3 RESULTS

### 3.1 BTZ slowed down the pathological process of AMR in a rat model and reduced the deposition of C4d and IgG

**FIGURE 1:**
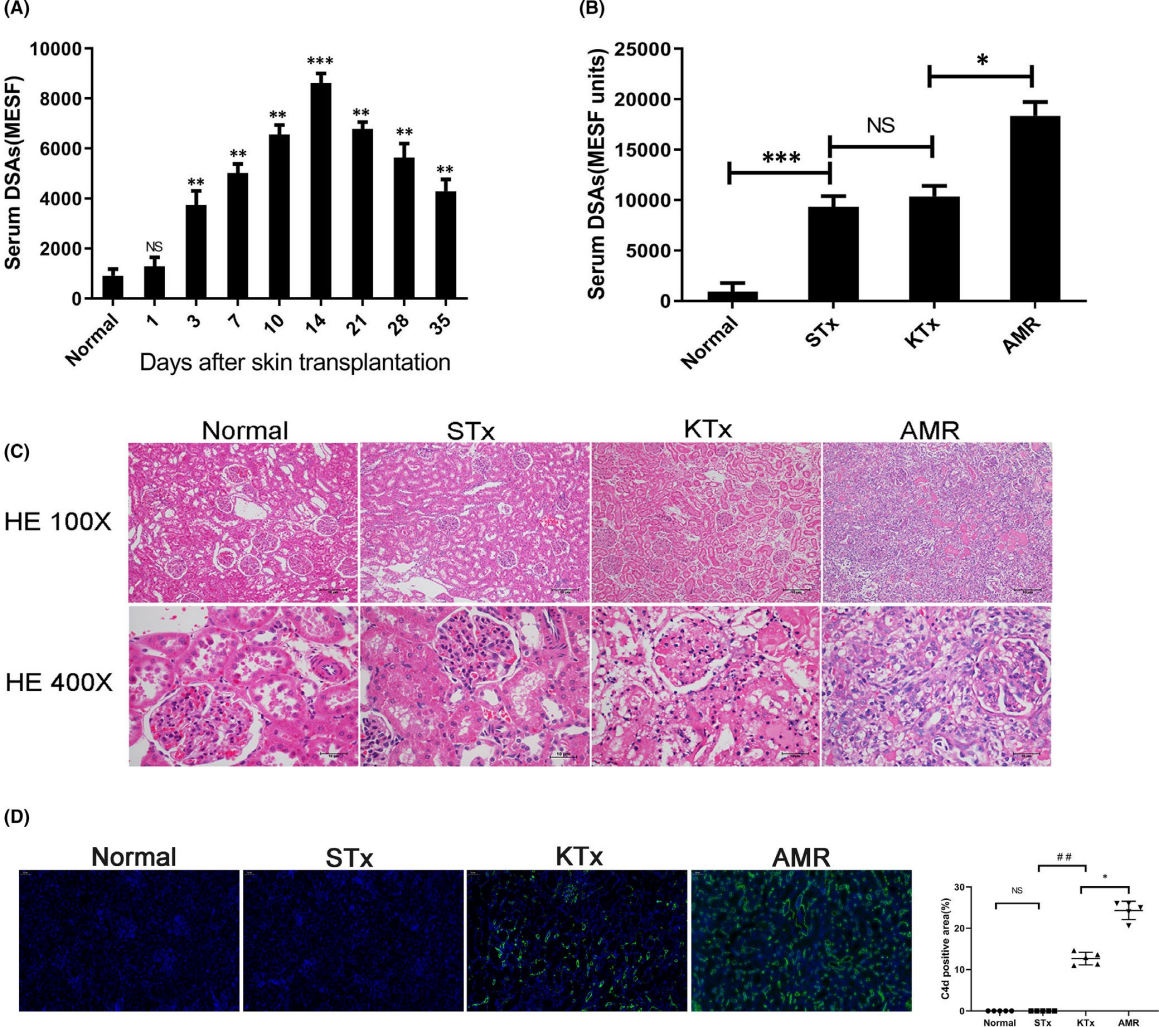
Constructing and identifying a rat model of AMR following kidney transplantation. Brown Norway (BN) rats were pre‐sensitized with Lewis (LEW) rats by skin transplantation to construct an antibody‐mediated rejection (AMR) model for kidney transplantation. Kidney grafts were harvested on day 4 after kidney transplantation. NS, no significance; *p < 0.05; ** *p* < 0.01; *** *p* < 0.001; ## *p* < 0.01. (A) Serum IgG level of BN rats after skin transplantation; mean fluorescence intensity (MFI) was converted to molecules of equivalent soluble fluorochrome (MESF) units. The level of donor‐specific antibodies (DSAs) reached peak level on day 14 after skin transplantation. (B) Serum IgG level of BN rats in the normal group, STx (skin transplantation) group, KTx (kidney transplantation) group and the AMR group. (C) Histopathology of the kidneys. Compared with the normal group, the STx group and the KTx group, the kidneys in the AMR group showed significant peritubular capillaries (PTC) inflammation and glomerulitis. (D) C4d staining of the kidneys. Compared with the normal group, STx group and the KTx group, C4d staining on PTCs was positive on day 4 in the AMR group following transplantation

**FIGURE 2:**
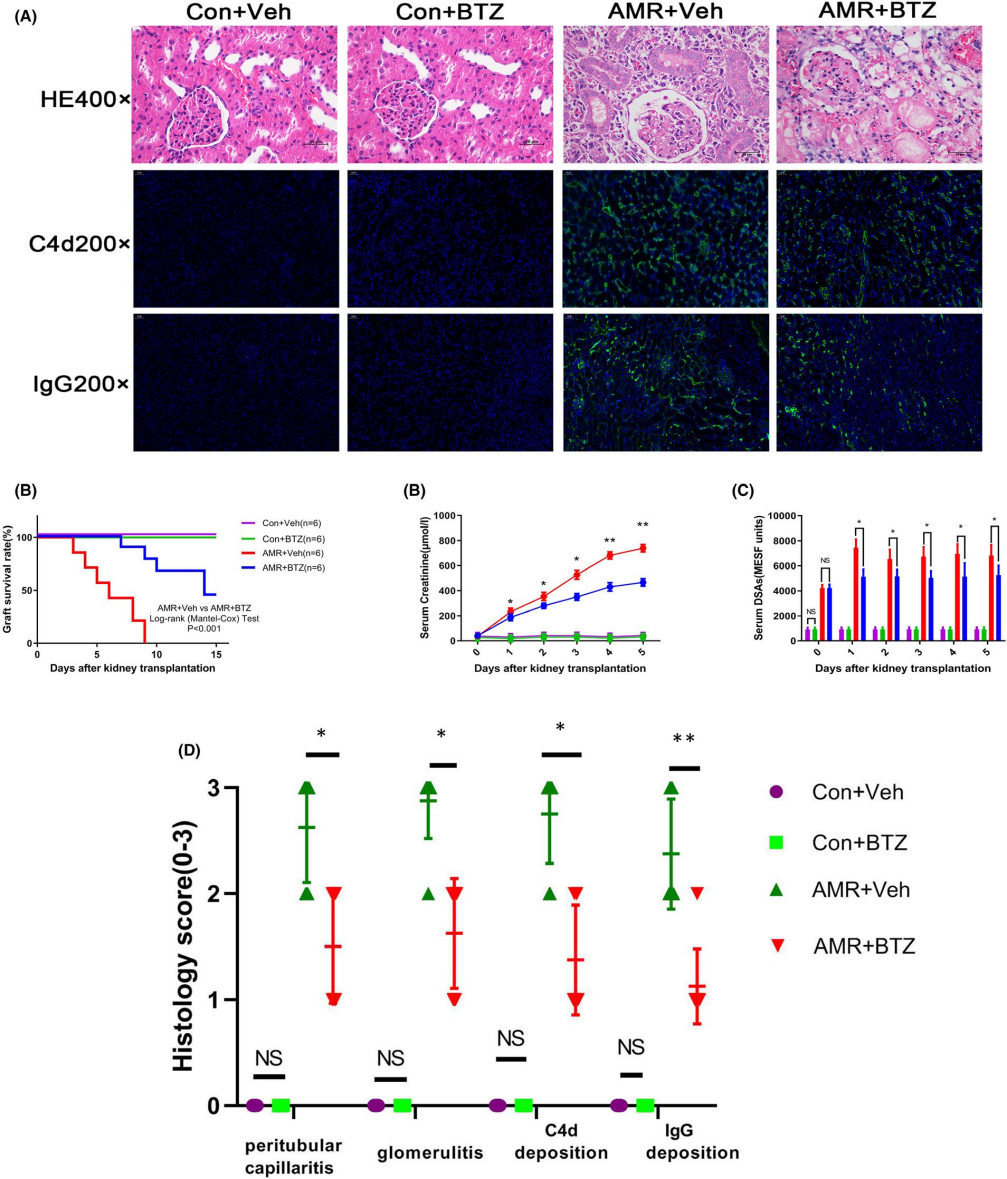
BTZ slowed down the pathological process of AMR, reduced serum level of DSAs and improved both renal function and graft survival in AMR rats. We harvested kidneys from the Con‐Veh group, the Con‐BTZ group, the AMR‐Veh group and the AMR‐BTZ group, on day 4 after transplantation. Kidneys were then stained with H&E and PAS. NS, no significance; * *p* < 0.05; ** *p* < 0.01; (A) The kidneys of the Con‐BTZ group showed similar pathological changes as the Con‐Veh group. Kidneys in the AMR‐Veh group developed serious PTC inflammation and glomerulitis following kidney transplantation. Compared with the AMR‐Veh group, the pathological process following kidney transplantation in the AMR‐BTZ group was significantly alleviated. There was no C4d and IgG staining on the PTCs in the Con‐Veh group and the Con‐BTZ group. However, there was intense and diffuse C4d and IgG staining on PTCs in the AMR‐Veh group; however, these effects were significantly alleviated in the AMR‐BTZ group. (B) The AMR‐BTZ group had a longer graft survival time compared with the AMR‐Veh group, with a mean survival time of 9.24 ± 4.01 vs 5.09 ± 2.26 days. The log‐rank (Mantel‐Cox) test revealed that BTZ significantly improve graft survival in a rat model of AMR. (C) Serum creatinine level in the AMR‐BTZ group was much higher than the Control group, although serum creatinine level was remarkably reduced by BTZ compared with the AMR‐Veh group (*p* < 0.05). (D) The serum level of DSAs was significantly reduced in AMR rats treated with BTZ when compared with rats with AMR but without BTZ treatment. (E) Histology scores of kidney according to Banff criteria

As shown in Figure 1A, the level of DSAs peaked on day 14 after skin transplantation. Kidneys were transplanted from donors to recipients when the level of DSAs reached the maximal level following skin transplantation. As shown in Figure 1B and Figure 1C, the AMR group exhibited typical PTC inflammation and glomerulitis, with a significant increase in the level of DSAs. Furthermore, PTCs were positive for C4d on day 4 in the AMR group after transplantation (Figure 1D). Collectively, these results indicated that the AMR model of rat kidney transplantation was successfully established. As shown in Figure 2A, the kidneys of the Con‐BTZ group exhibited similar pathological changes to those in the Con‐Veh group. However, kidneys in the AMR‐Veh group exhibited far more extensive and significant PTC inflammation and glomerulitis compared with the AMR‐BTZ group, thus indicating that the pathological process associated with kidney transplantation was much reduced in the AMR‐BTZ group. Furthermore, C4d and IgG staining on the PTCs was intense and diffuse in the AMR‐Veh group; following BTZ treatment, these symptoms became milder and less concentrated. In addition, C4d and IgG staining showed that BTZ also significantly reduced the deposition of C4d and IgG. Collectively, these results demonstrated that BTZ can slow down the pathological process of AMR.

### 3.2 BTZ reduced the serum level of DSAs and improved renal function and graft survival in a rat model of AMR rats

As shown in Figure 2B, there was no significant difference in graft survival time when comparing the Con‐Veh group with the Con‐BTZ group. However, graft survival time was significantly longer in the AMR‐BTZ group than in the AMR‐Veh group (9.24 ± 4.01 vs 5.09 ± 2.26 days; *p* < 0.001, log‐rank (Mantel‐Cox) Test). In addition, serum creatinine level in rats from the AMR‐BTZ group was significantly higher than in the Con‐Veh group, but improved significantly in response to BTZ treatment from day 1 when compared with the AMR‐Veh group (*p* < 0.05) (Figure 2C). Most importantly, the level of DSAs decreased significantly in AMR rats following BTZ treatment (*p* < 0.05) (Figure 2D). Collectively, these results demonstrated that BTZ could reduce the level of serum DSAs, improve renal function and prolong graft survival in a rat model of AMR. Two pathologists scored the pathology, respectively, according to the Banff criteria and synthesize the score.

### 3.3 BTZ reduced the number of B cell and plasma cell in peripheral blood and the transplanted kidney

**FIGURE 3:**
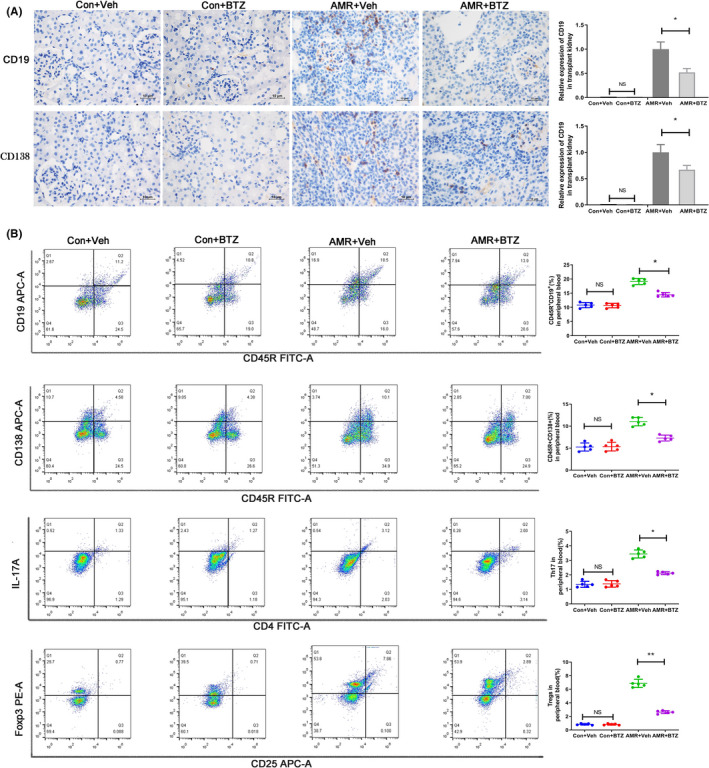
BTZ reduced the number of B cell and plasma cell in peripheral blood and transplanted kidneys, and significantly increased the level of Treg in rats with AMR. We investigated the infiltration of B cell and plasma cell by immunohistochemistry. We also used flow cytometry to investigate peripheral blood for B cell (CD45R+, CD19+), plasma cell (CD45R**^−^,** CD138^+^), Treg (CD4^+^, CD25^+^, FOXP3^+^) and Th17 (CD4^+^, IL‐17A^+^). NS, no significance; * *p* < 0.05; ** *p* < 0.01. (A) There was a significant reduction of B cell and plasma cell in the AMR‐BTZ group compared with the AMR‐Veh group. (B) There was no significant difference in the numbers of B cell and plasma cell when compared between the Con‐Veh group and the Con‐BTZ group. However, the numbers of B cell and plasma cell were significantly reduced in the AMR‐BTZ group compared with the AMR‐Veh group. Compared with the AMR‐Veh group, the ratio of Treg was increased and the ratio of Th17 was decreased in the AMR‐BTZ group (*p* < 0.05)

Immunohistochemical analysis of the transplanted kidney on day 4 after kidney transplantation showed that the level of B and plasma cell infiltration was similar when comparing the Con‐Veh group with the Con‐BTZ group. However, the number of cell was significantly reduced in the AMR‐BTZ group compared with the AMR‐Veh group (*p* < 0.05) (Figure 3A). Flow cytometry, carried out on peripheral blood acquired on day 4 after kidney transplantation, revealed that there were no significant differences in the number of B cell (CD45R^+^CD19^+^) and plasma cell (CD45R^+^CD138^+^) when comparing the Con‐Veh group with Con‐BTZ group. However, the number of B cell and plasma cell was significantly reduced after BTZ treatment (*p* < 0.05) (Figure 3B). In conclusion, BTZ reduced the number of B cell and plasma cell in a rat model of AMR.

### 3.4 BTZ modulated the ratio of Th17/Treg in rats with AMR

**FIGURE 4:**
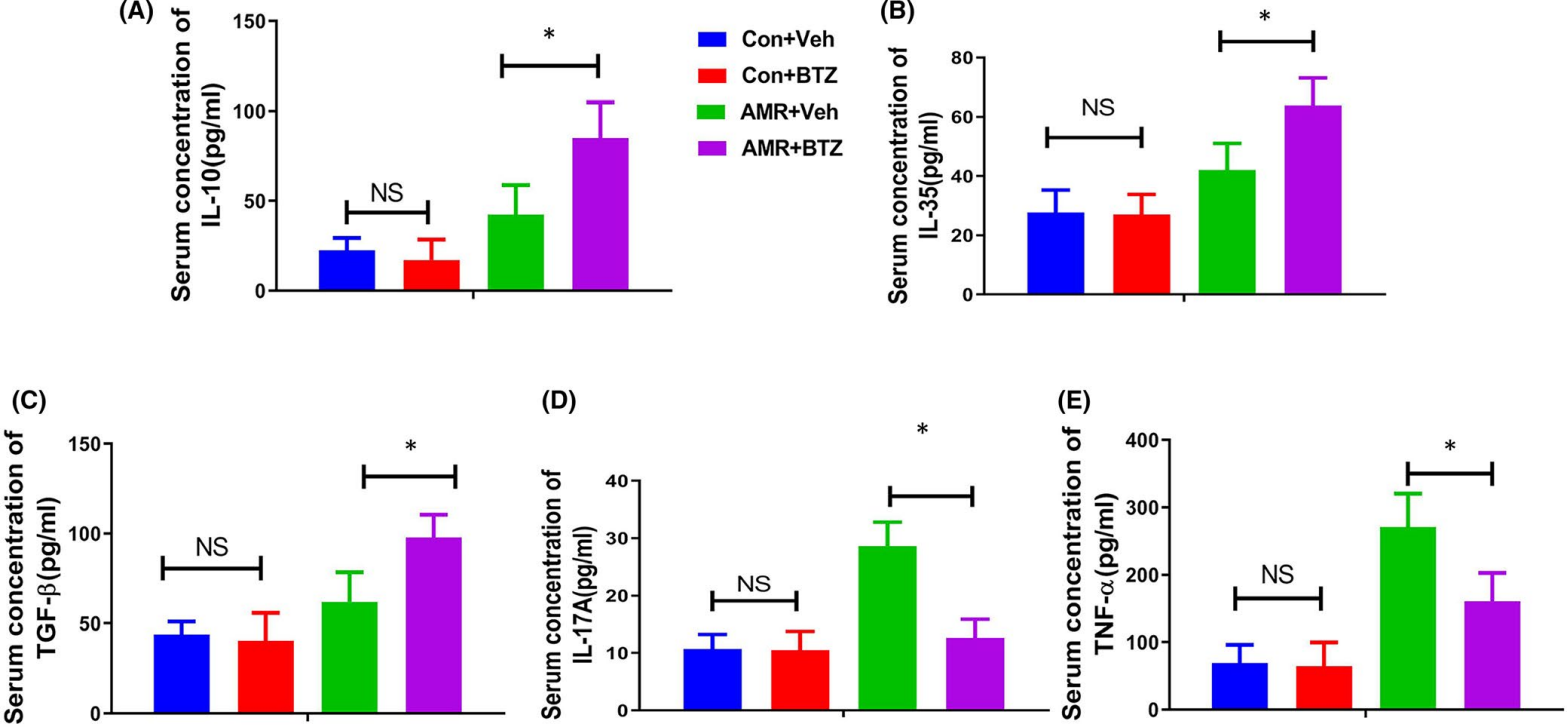
BTZ significantly increased the level of anti‐inflammatory cytokines and decreased the level of pro‐inflammatory cytokines. Serum cytokines were determined by ELISA. NS, no significance; * *p* < 0.05. (A) Serum concentration of IL‐10. (B) Serum concentration of IL‐35. (C) Serum concentration of TGF‐β. (D) Serum concentration of IL‐17A. (E) Serum concentration of TNF‐α

Peripheral blood samples were taken on day 4 after kidney transplantation. We then detected Treg (CD4^+^CD25^+^FOXP3^+^) and Th17 (CD4^+^IL‐17A^+^) by flow cytometry. There was no significant difference in the ratio of Treg and Th17 when comparing the Con‐Veh group with the Con‐BTZ group. However, the ratio of Treg and Th17 in the AMR‐BTZ group showed significant changes when compared with the AMR‐Veh group; in the AMR‐BTZ group, BTZ treatment led to a significant increase in the level of Treg but a significant reduction in Th17 (*p* < 0.05) (Figure 3B). Furthermore, BTZ treatment also resulted in a significant increase in the level of several anti‐inflammatory cytokines, including IL‐10, IL‐35 and TGF‐β (*p* < 0.05) (Figure 4A–4C). However, BTZ treatment caused a significant reduction in pro‐inflammatory cytokines (IL‐17A and TNF‐α) (*p* < 0.05) (Figure 4D,4E). Therefore, we hypothesized that BTZ regulates the immune status of the body by modulated the ratio of Th17/Treg.

### 3.5 BTZ facilitates the differentiation of Treg by facilitating the expression of Atg5

**FIGURE 5:**
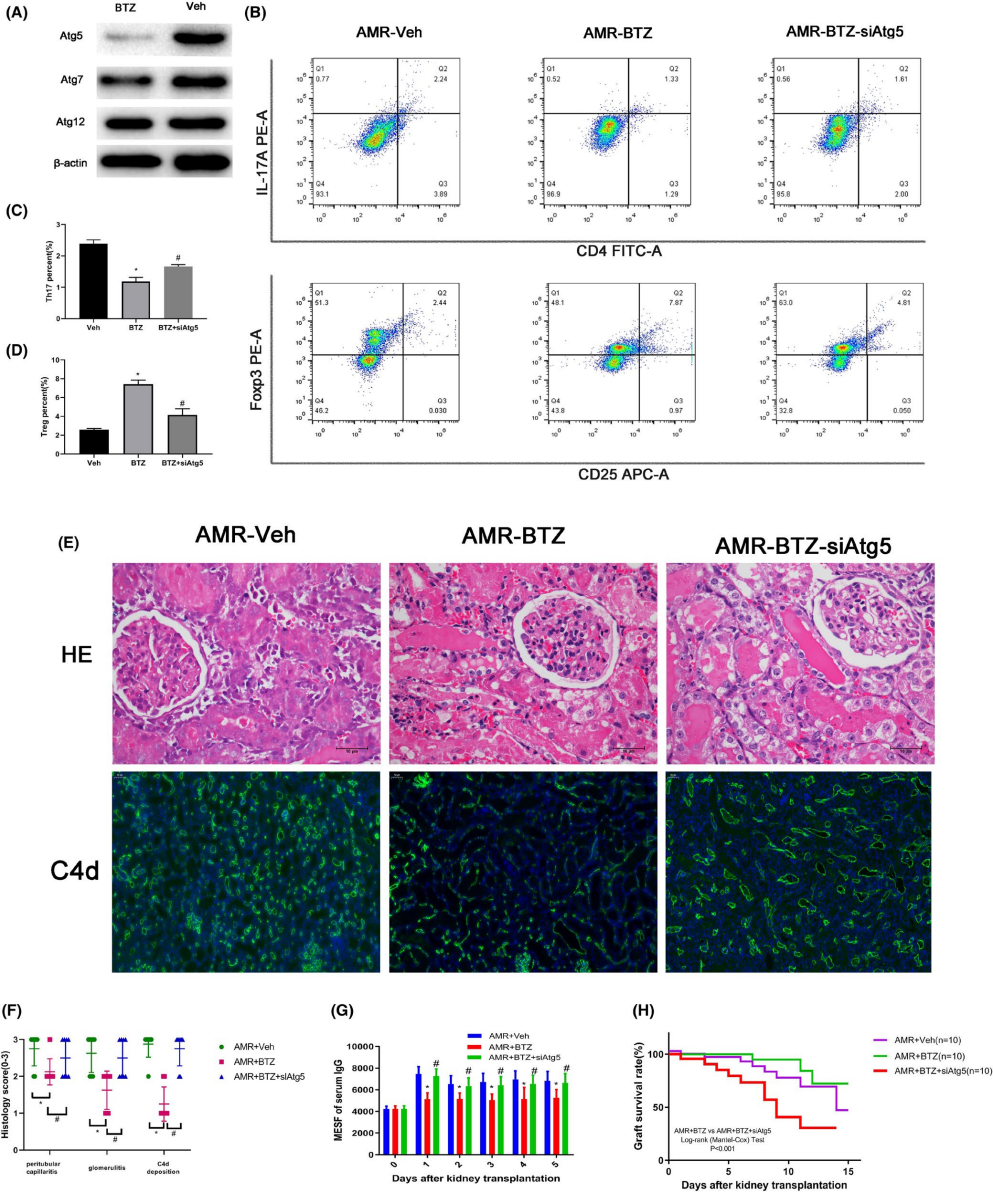
BTZ modulating the differentiation of Th17/Treg by facilitating the expression of Atg5. * *p* < 0.5; # *p* < 0.05. (A) Atg5, Atg7, Atg12 expression in the AMR‐Veh group and the AMR‐BTZ group. (B‐D) The relative proportions of Treg and Th17 in the AMR‐Veh group, the AMR‐BTZ group and the AMR‐BTZ‐siAtg5 group. (E) HE staining and C4d staining of kidneys in the AMR‐Veh group, the AMR‐BTZ group, and the AMR‐BTZ‐siAtg5 group. (F) Histology score of kidney according to Banff criteria. (G) The serum level of DSAs in the AMR‐Veh group, the AMR‐BTZ group and the AMR‐BTZ‐siAtg5 group. (H) The survival curves of rats in the AMR‐Veh group, the AMR‐BTZ group and the AMR‐BTZ‐siAtg5 group

Since BTZ was found to alleviate AMR by modulating the expression of Th17/Treg, we next explored the potential mechanism underlying the action of BTZ. Atg5 is a well‐known gene related to autophagy and has been widely proven to facilitate the differentiation of Treg.^26^ First, we isolated total T cells from peripheral blood samples and then determined the expression of Atg5. We demonstrated that BTZ induced the significant upregulation of Atg5 in T cell, thus indicating that BTZ might modulate the differentiation of Th17/Treg by facilitating the expression of Atg5 (Figure 5A). Next, we isolated CD4 T cell from BN rat and cultured these cells in vitro. Then, the cells were treated BTZ or combined with an Atg5 siRNA‐lentivirus. We demonstrated that BTZ treatment facilitated the differentiation of Treg and blocked the differentiation of Th17. However, Atg5 silencing reversed this effect (Figure 5B–5D). Notably, the expression of Th17/Treg was related to Atg5. Furthermore, our results demonstrated that Atg5 silencing reversed the alleviation of AMR induced by BTZ treatment, including reduced DSAs of serum, C4d deposition in peritubular capillaries and alleviated the pathologic process (Figure 5E–5G), and that the prolonged survive time induced by BTZ treatment was also reversed by Atg5 silencing (Figure 5H). Collectively, these results demonstrated that BTZ might alleviate AMR by facilitating Atg5 expression to modulating the differentiation of Th17/Treg.

## 4 DISCUSSION

In this study, we constructed and verified a rat model of AMR following kidney transplantation. On this basis, we used model to investigate the effect of BTZ on AMR. We found that BTZ slowed down the pathological process of AMR and reduced the deposition of C4d and IgG. In addition, BTZ reduced the number of B cell and plasma cell, and improved both renal function and graft survival in AMR rats. Our results also found that BTZ increased the number of Treg and significantly reduced the proportion of Th17 (*p* < 0.05).

Relevant studies have shown that BTZ plays an important role in the chronic rejection.^27^, ^28^, ^29^ However, previous researches have not fully explained the effect and mechanism of BTZ on AMR. A randomized trial of BTZ in AMR was conducted and the result showed that BTZ did not have good effects for AMR, despite significant toxicity.^30^ However, in one clinical trial, the mid‐term observation of primary BTZ‐based treatment for AMR patients showed its non‐inferiority and acceptable safety profile when compared to previously proposed regimens.^30^ In another clinical trial, BTZ could stabilize glomerular filtration rate for half a year in paediatric kidney transplant recipients with AMR.^31^ In the present study, we founded that BTZ significantly reduced the level of DSAs and slowed down the pathological process of AMR in a rat model. Most importantly, we validated the role of BTZ in AMR and initially explored the mechanism of BTZ on AMR.

In the present study, we also found that BTZ treatment led to an increase in the level of Treg but a significant reduction in Th17. Consistently, we found that the level of anti‐inflammatory cytokines (IL‐10, IL‐35 and TGF‐β) was significantly increased after BTZ treatment, while level of pro‐inflammatory cytokines (IL‐17A and TNF‐α) were significantly reduced in response to BTZ treatment. Next, we carried out a preliminary exploration of the mechanisms underlying the action of BTZ on AMR. We found that BTZ might alleviate AMR by facilitating the expression of Atg5 to modulating the differentiation of Th17/Treg. Atg5 is a well‐known autophagy gene and the silencing of this gene is known to activate T cell and fail to upregulate Foxp3 when compared to wild‐type cells.^32^ In addition, Wei et al. previously showed that Atg5 or Atg7‐deficient Treg cell showed increased level of apoptosis and readily lost their ability to express the Foxp3 transcription factor, particularly after activation.^33^ Carriche et al. revealed that activated T cell lacking the Atg5 failed to upregulate the expression of Foxp3.^32^

BTZ was the first clinical proteasome inhibitor and has been approved for the treatment of multiple myeloma. BTZ also is a relatively new medication in the transplantation field, and while there is no research providing a clear indication for its indication in transplant. Although additional clinical trials are needed to support BTZ as an effective alternative medication for desensitization with fairly good short‐term success rate, its utility for treating AMR appears to be more uncertain. In this research, we investigated the effect of BTZ on AMR following kidney transplantation using a rat AMR model. Our results demonstrated BTZ alleviated AMR by reducing antibody deposition in tissues and serum. However, these effects were reversed when Atg5 was silenced. Our findings provide a new theoretical basis for the prevention and treatment of AMR after renal transplantation.

In this research, we only found that BTZ modulating the differentiation of Th17/Treg and alleviate AMR through Atg5. There was no direct inhibition of Treg to observe the effect of BTZ on AMR. We found that BTZ can alleviate AMR by regulating Atg5 expression rather than induce immune tolerance. Furthermore, our discovery is just one way that BTZ act on AMR, and indirectly proved that BTZ alleviates AMR may modulate the proportion of Th17/Treg. Therefore, more experiments are needed to verify our hypothesis that BTZ inhibits AMR by regulating Th17/Treg.

In summary, we found that BTZ was able to reduce the level of DSAs and slow down the pathological process of AMR in rat model. Atg5 plays a vital role in the mechanism of BTZ for AMR and Atg5 may alleviate AMR by modulating the expression of Th17/Treg. This research provides support for the inhibition of AMR by BTZ and Atg5 siliencing may be an effective therapeutic regimen for AMR.

## CONFLICT OF INTEREST

The authors confirm that there are no conflicts of interest.

## AUTHOR CONTRIBUTIONS

**Hong Cheng:** Conceptualization (lead); Methodology (lead); Writing‐original draft (lead). **Bin Xu:** Methodology (equal); Resources (lead); Supervision (lead); Writing‐review & editing (equal). **Lijie Zhang:** Project administration (equal); Software (equal); Validation (equal); Visualization (equal). **Yi Wang:** Conceptualization (equal); Data curation (equal); Investigation (equal); Software (equal). **Ming Chen:** Data curation (equal); Formal analysis (equal); Funding acquisition (equal); Investigation (lead); Project administration (equal); Visualization (equal); Writing‐review & editing (equal). **Shuqiu Chen:** Conceptualization (lead); Data curation (lead); Funding acquisition (lead).

## Data Availability

The data sets used and analysed during the current study are available from the corresponding author on reasonable request.
